# Structural Transitions in Ge_2_Sb_2_Te_5_ Phase Change Memory Thin Films Induced by Nanosecond UV Optical Pulses

**DOI:** 10.3390/ma13092082

**Published:** 2020-05-01

**Authors:** Mario Behrens, Andriy Lotnyk, Hagen Bryja, Jürgen W. Gerlach, Bernd Rauschenbach

**Affiliations:** 1 Department of Precision Surfaces, Leibniz Institute of Surface Engineering (IOM), Permoserstr 15, 04318 Leipzig, Germany; hagen.bryja@iom-leipzig.de (H.B.); juergen.gerlach@iom-leipzig.de (J.W.G.); bernd.rauschenbach@iom-leipzig.de (B.R.); 2 Laboratory of Infrared Materials and Devices, The Research Institute of Advanced Technologies, Ningbo University, Ningbo 315211, China

**Keywords:** phase change materials, phase transitions, thin films, optical switching, electron microscopy

## Abstract

Ge-Sb-Te-based phase change memory alloys have recently attracted a lot of attention due to their promising applications in the fields of photonics, non-volatile data storage, and neuromorphic computing. Of particular interest is the understanding of the structural changes and underlying mechanisms induced by short optical pulses. This work reports on structural changes induced by single nanosecond UV laser pulses in amorphous and epitaxial Ge_2_Sb_2_Te_5_ (GST) thin films. The phase changes within the thin films are studied by a combined approach using X-ray diffraction and transmission electron microscopy. The results reveal different phase transitions such as crystalline-to-amorphous phase changes, interface assisted crystallization of the cubic GST phase and structural transformations within crystalline phases. In particular, it is found that crystalline interfaces serve as crystallization templates for epitaxial formation of metastable cubic GST phase upon phase transitions. By varying the laser fluence, GST thin films consisting of multiple phases and different amorphous to crystalline volume ratios can be achieved in this approach, offering a possibility of multilevel data storage and realization of memory devices with very low resistance drift. In addition, this work demonstrates amorphization and crystallization of GST thin films by using only one UV laser with one single pulse duration and one wavelength. Overall, the presented results offer new perspectives on switching pathways in Ge-Sb-Te-based materials and show the potential of epitaxial Ge-Sb-Te thin films for applications in advanced phase change memory concepts.

## 1. Introduction

The working principle of conventional phase change memory is based on fast reversible phase changes between crystalline and amorphous phases of Ge-Sb-Te materials [1,2,3]. For information storage, a phase change memory device uses a large contrast either in electrical resistance between the amorphous phase (high-resistance phase, reset state) and the crystalline phase (low-resistances phase, set state) or in optical reflectivity between the amorphous phase (low reflectivity phase) and the crystalline phase (high reflectivity phase). Resetting a Ge-Sb-Te-based memory cell is achieved by applying a high intensity electrical or optical pulse [4,5,6]. This process leads to amorphization via melting and subsequent fast quenching of the phase change alloy [7]. However, Ge-Sb-Te alloys are poor glass formers [8,9]. Consequently, high cooling rates are required to suppress the recrystallization of the alloys during the erase process [10,11,12]. Contrary, the set process is accomplished by applying either electrical or optical pulses with lower intensity and longer duration, resulting in an amorphous-to-crystalline phase transition [13,14]. Due to intrinsic features of Ge-Sb-Te alloys, a main challenge in material science is the optimization of memory writing times (set process), which are limited by the crystallization kinetics of Ge-Sb-Te alloys. In order to increase the crystallization rates, several strategies were proposed, including doping of Ge-Sb-Te materials, pre-crystallization of an amorphous matrix or using Ge-Sb-Te-based heterostructures [15,16,17,18,19]. Being a nucleation-dominant material, improvements in crystallization speed can, however, be achieved by taking advantage of interface assisted crystal growth, which is considered to occur in nanoscale phase change memory cells [20]. This approach will overcome the time-lag originating from the incubation period during nucleation [21,22]. Moreover, due to the increasing interest of Ge-Sb-Te-based materials for photonic applications [3,23,24,25], a detailed understanding of phase change processes induced by short optical pulses in those alloys is required.

Thus, this work aims to study structural transitions in amorphous and layered epitaxial Ge_2_Sb_2_Te_5_ (GST) thin films induced by single UV ns-laser pulse in order to explore possible switching pathways, which might represent optimized transitions in terms of entropic loss, switching times, and energy consumption.

## 2. Materials and Methods

Amorphous and epitaxial GST thin films were deposited on Si (111) substrates by pulsed laser deposition (PLD) [26]. A KrF excimer laser (Coherent Inc., Santa Clara, CA, USA) with 248-nm wavelength, 20-ns pulse duration and 20-Hz repetition rate was used to ablate a GST compound target. The base pressure of the deposition chamber was 1.3 × 10^−8^ mbar and a constant Ar gas flow was introduced to moderate the kinetic energy of the ablated species. The as-grown films were finally capped with an optically transparent LaAlO_3-x_ (LAO) protective layer of about 5 nm thickness to protect the GST thin films against oxidation. The structure of as-grown and laser irradiated thin films was studied ex-situ by X-ray diffraction (XRD) measurements (Cu Kα radiation, parallel beam geometry). In order to induce structural transitions, the GST thin films were irradiated with a single laser pulse at a wavelength of 248 nm and pulse duration of 20 ns. For the optical switching experiments, the same laser was used as for the PLD growth of GST thin films. The temporal pulse shape was Top Hat. The size of the laser beam on the surface of the thin films was 0.5 cm × 1 cm. The area of X-ray probing was smaller than the area of GST thin films irradiated by the UV laser.

The microstructure of GST thin films was investigated in a probe Cs-corrected Titan^3^ G^2^ 60–300 transmission electron microscope operating at 300 kV accelerating voltage. Bright-field transmission electron microscopy (BF-TEM) and high-resolution transmission electron microscopy (HRTEM) images as well as selected area electron diffraction (SAED) patterns were acquired using a Gatan CCD camera (Gatan Inc., Pleasanton, CA, USA). For high-resolution scanning TEM (HRSTEM), a probe forming annular aperture of 25 mrad was used and all images were recorded with a high-angle annular dark-field (HAADF) detector using annular ranges of 80–200 mrad. The preparation of TEM specimens was performed by a combination of focused high- and low-energy ion beam milling. 

## 3. Results and Discussion

### 3.1. Crystalline-to-Amorphous Phase Transitions

**Figure 1:**
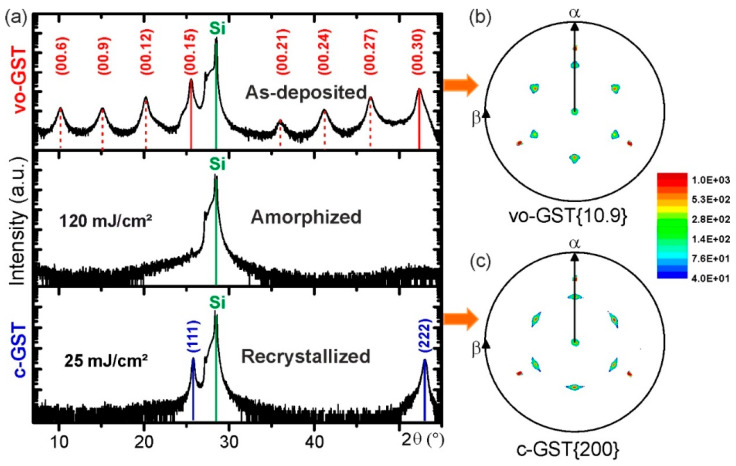
(**a**) XRD patterns of reversible crystalline-to-amorphous phase transitions in an epitaxial GST thin film induced by ns-laser irradiation. The upper XRD pattern originates from the as-deposited vo-GST thin film. The middle pattern corresponds to the thin film after applying a single ns-laser pulse with a fluence of 120 mJ/cm^2^ leading to amorphization. The bottom pattern is related to the thin film after recrystallization by applying a single ns-laser pulse with a fluence of 25 mJ/cm^2^. The diffractogram of as-deposited vo-GST thin film shows superstructure reflections (vacancy layer peaks) marked by red dashed lines beside the main reflections marked by red continuous lines. The continuous blue and green lines mark (111) lattice planes in the c-GST and Si substrate, respectively. (**b**) and (**c**) XRD pole figure measurements of as-deposited and recrystallized GST thin film.

First, crystalline-to-amorphous phase transitions induced by single ns-laser pulse irradiation of epitaxial GST thin films with vacancy ordered metastable phase (vo-GST) were studied. Figure 1a shows ω−2θ diffraction patterns of a GST thin film at three different stages of the switching process: (1) initial crystalline state (upper pattern), (2) melt-quenched amorphous state (middle pattern) and (3) recrystallized state (lower pattern). The upper XRD pattern of Figure 1a confirms the overall structure of as-deposited GST thin films. The XRD pattern shows the appearance of superstructure reflections, pointing out the regularly spaced vacancy layers (VLs) in the thin film. The film is characterized by a continuous cubic stacking sequence of cation and anion atomic layers. Similar vo-GST structure was reported by other groups [27,28,29,30]. The full description of vo-GST phase can be found in reference [1]. Briefly, the vo-GST phase belongs to the trigonal crystal system (P−3m1 space group, a = 0.426 nm and c = 5.126 nm) and represents a superstructure of the rock-salt type with the cubic stacking of Te layers across the VLs. Although closely related, the vo-GST phase differs clearly from the stable trigonal phase of GST (t-GST), which possesses a hexagonal stacking sequence of Te atomic layers across the van der Waals (vdW)-like gaps [26,31,32]. Further details on the texture of the vo-GST thin films were obtained from XRD pole figure measurements. Figure 1b depicts an in-plane pole figure of the GST {200} reflection of the as-deposited GST thin film and points out the previously observed epitaxial relationship between the substrate and the vo-GST thin film. Furthermore, the presence of 180° rotational twin domains is indicated by the occurrence of 2 × 3 instead of 1 × 3 pole density maxima in the pole figure.

By applying a single ns-laser pulse with a fluence of 120 mJ/cm^2^, the epitaxial vo-GST thin film undergoes a phase transition to an amorphous GST (a-GST) thin film, which becomes apparent by the corresponding ω−2θ pattern showing only substrate contributions (Figure 1a). However, that does not necessarily imply that the complete short-range order of atoms in the melt-quenched GST thin film is lost. Instead, a certain short-range order could be preserved in accordance with various structural models proposing residual structural motifs in the melt-quenched state [33,34,35,36].

In the following step, the amorphized GST thin film is recrystallized by applying a single ns-laser pulse with a fluence of 25 mJ/cm^2^. The bottom ω−2θ XRD pattern of Figure 1a shows the recrystallized GST thin film and reveals the formation of a metastable GST cubic phase (c-GST) with a random distribution of vacancies. The formation of c-GST is evident from the appearance of c-GST (111) and c-GST (222) reflections, showing epitaxial recrystallization of the c-GST phase in addition (Figure 1c). The corresponding XRD pole figure measurement in Figure 1c confirms an epitaxial relationship between the Si (111) substrate and the recrystallized c-GST thin film, which is equivalent to the initial epitaxial relationship of the as-deposited vo-GST thin film. Consequently, during the switching process the initial epitaxial nature of the GST film is restored. The vacancy ordering, however, is lost, since the epitaxial GST recrystallizes into the vacancy disordered metastable phase. Furthermore, it is found that a repetition of the switching procedure (at least two cycles) results again in the recrystallization of the epitaxial cubic phase, where the Si (111) acts as an epitaxial template for the formation of c-GST. Hence, it is possible to induce reversible structural transitions between epitaxial and amorphous GST thin films. This might offer advantages like a larger property contrast and a more precise control of the contrast due to high-quality epitaxial thin films (compared to switching between amorphous and polycrystalline GST thin films). It should be noted that UV laser irradiation might cause surface damage of GST thin films after multiple cycles. However, it was dependent on the applied laser fluence. A high laser fluence always caused surface damage while this effect can be minimized by selecting an optimized laser fluence.

**Figure 2:**
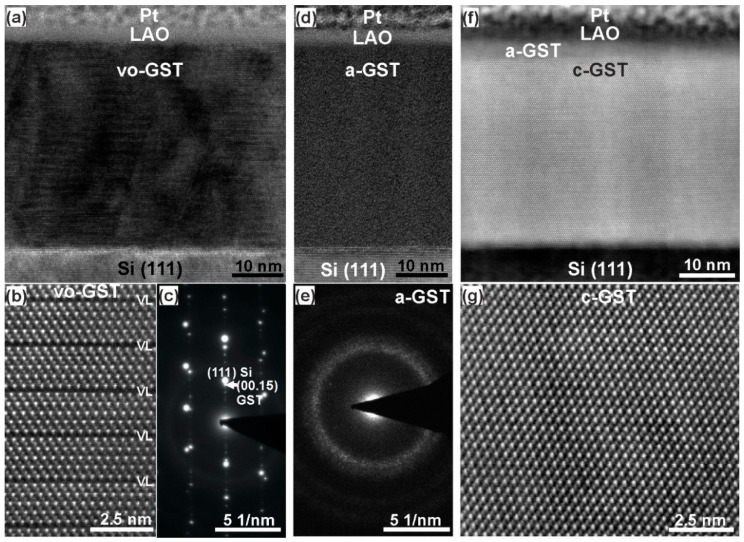
(**a**) BF-TEM and (**b**) HAADF-HRSTEM images of an as-deposited vo-GST thin film. The dark lines in (**b**) are VLs. The bright dots in (**b**) are Te atomic columns whereas the darker dots are mixed Ge/Sb atomic columns. (**c**) SAED pattern showing 5-fold periodicity in the as grown thin film confirming the presence of periodically spaced VLs. (**d**) BF-TEM image and (**e**) SAED pattern of amorphized GST thin film. (**f**) and (**g**) HAADF-HRSTEM images of recrystallized GST thin film, showing epitaxial formation of c-GST phase. Viewing direction is vo-GST [11,12,13,14,15,16,17,18,19,20] || Si [1,2,3,4,5,6,7,8,9,10] || c-GST [1,2,3,4,5,6,7,8,9,10].

Further insights into the microstructure of such optically switched GST thin films were gained from TEM measurements. Figure 2 shows BF-TEM and HRTEM images and corresponding SAED patterns of as-grown vo-GST (Figure 2a–c), the amorphized (Figure 2d,e) and the recrystallized (Figure 2f,g) thin films, respectively. Clearly, the TEM micrographs verify the reversible crystalline-to-amorphous phase transitions and the recovery of epitaxial relationship during the recrystallization process. However, as revealed by the XRD analysis, Figure 2f additionally shows that besides c-GST, a thin layer of a-GST has formed at the top of the thin film. This indicates an incomplete phase transition due to higher cooling rates caused by the LAO protective layer (see more discussions later). Nevertheless, the epitaxial switching mechanism is confirmed by these TEM results.

### 3.2. Influence of Interfaces on Crystallization Process

**Figure 3:**
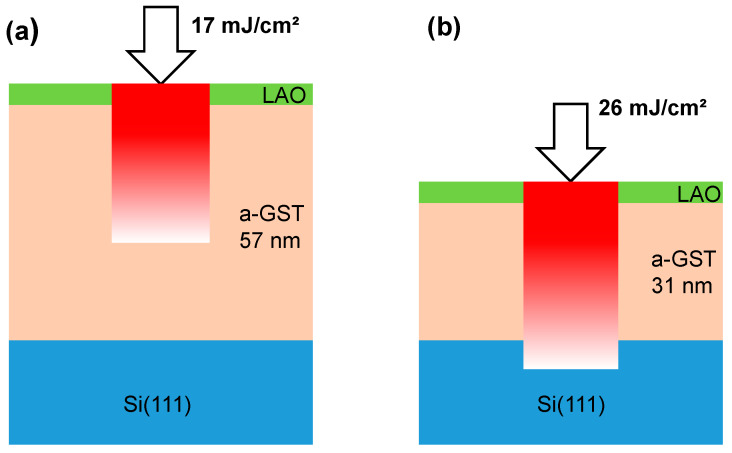
Scheme for the investigation of the effect of interfaces on the crystallization of GST thin films during ns-laser irradiation. The figure shows approximately the propagation of the laser beam in the GST thin films. (**a**) Illustration of the experiment on UV laser irradiation of an amorphous GST thin film of thickness 57 nm by a ns-laser pulse with a fluence of 17 mJ/cm^2^; (**b**) Representation of the experiment on UV laser irradiation of an amorphous GST thin film of thickness 31 nm by a ns-laser pulse with a fluence of 26 mJ/cm^2^.

At this point, the question arises how the structural information leading to the formation of epitaxial c-GST is retrieved during the observed recrystallization process. Thus far, the rare literature on the underlying mechanism of switching epitaxial thin films is not conclusive [37,38,39]. Therefore, additional experiments were performed to examine whether the recrystallization process starts at the a-GST/Si (111) interface with the substrate acting as a crystallization template and epitaxially proceeds towards the top of the thin film. In detail, amorphous GST thin films of 57 and 31 nm thicknesses were deposited on Si (111) substrates without native oxide. Then, the 57-nm thick GST thin film was irradiated by a ns-laser pulse with a fluence of 17 mJ/cm^2^, whereas the 31-nm thick film was irradiated by a laser pulse with a fluence of 26 mJ/cm^2^. This experimental procedure is schematically illustrated in Figure 3. The intention of the additional experiments was put on the comparison of the crystallization behavior of GST thin films starting from the Si (111) substrate and the surface of the GST thin film. In the first case (Figure 3a), no laser intensity produced by the laser irradiation at low fluence reaches the substrate, since the laser light is significantly absorbed by the thicker film and the crystallization is expected to start from the surface of GST thin film. The absorption coefficient of GST in the UV region is ~10^6^ cm^−1^. In the case of the thinner film (Figure 3b), a significant laser intensity produced by laser irradiation at high fluence reaches the substrate and the crystallization is expected to start from the substrate.

**Figure 4:**
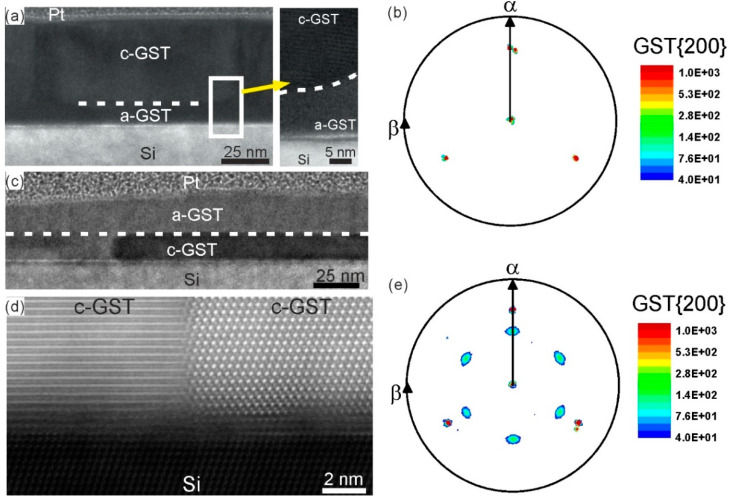
(**a**) BF-TEM image of a-GST thin film irradiated by a single ns-laser pulse with a fluence of 17 mJ/cm^2^. The interface between the crystalline and the amorphous region is indicated by a dashed line. (**b**) XRD pole figure of the c-GST {200} reflections exhibiting only the pole density maxima contributions from the Si substrate. (**c**) BF-TEM image of a-GST thin film irradiated by a single ns-laser pulse with a fluence of 26 mJ/cm^2^. A dashed line indicates the interface between the crystalline and the amorphous region. (**d**) HAADF-HRSTEM image of the GST-Si (111) interface formed during crystallization of a-GST. (**e**) XRD pole figure of the c-GST {200} reflections exhibiting contributions from both the epitaxially recrystallized GST film and the Si substrate.

Figure 4a shows a cross-sectional TEM image of the 57-nm thick amorphous GST thin film irradiated by a low intensity ns-laser single pulse with a fluence of 17 mJ/cm^2^. The image reveals the formation of GST grains extending from the film surface towards the substrate. In addition, a thin layer of a-GST between the substrate and the crystalline GST has remained. Hence, the crystallites are not in direct contact with the substrate surface and they are randomly oriented, which is confirmed by the corresponding pole figure measurement of the GST {200} reflections (Figure 4b). In contrast, Figure 4c,d show TEM images of the 31-nm thick GST thin film irradiated by the ns-laser with a fluence of 26 mJ/cm^2^. The TEM results reveal an epitaxial relationship between the crystallized c-GST structure and the Si (111) substrate. Here, the GST grains are in direct contact with the substrate. This is a strong evidence for a laser induced epitaxial crystallization starting at the substrate surface. Thus, in this case, the relation between laser fluence and the thin film thickness leads to a sufficient crystallization temperature at the Si/a-GST interface, where an epitaxial crystallization process starts at the substrate surface and propagates towards the thin film surface. It should be noted that epitaxial recrystallization of GST thin films grown by molecular beam epitaxy on Si (111) substrates can be also achieved by using femtosecond laser pulses [40].

The results in Figure 4 clearly demonstrate that epitaxial c-GST layers can be obtained by laser irradiation of amorphous GST grown on crystalline Si (111) substrates. Hence, the epitaxial formation of c-GST is achieved on time scales ranging from minutes and hours, as in the case of epitaxial thin film deposition, down to a few nanoseconds, as in the case of ns-laser annealing where fast crystal growth rates are involved.

**Figure 5:**
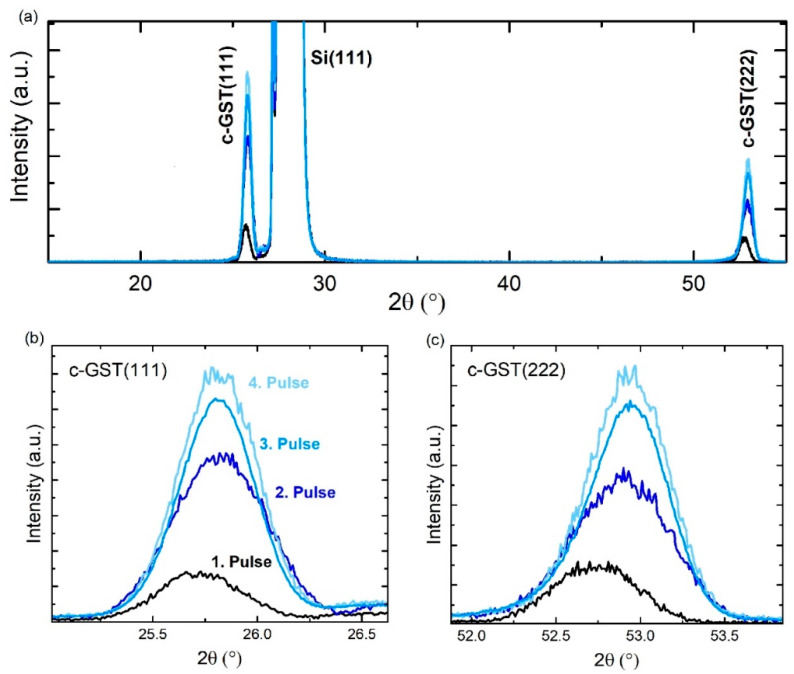
(**a**) XRD ω−2θ patterns of a GST thin film after application of successive single ns-laser pulses at 21 mJ/cm^2^ fluence. XRD patterns in the vicinity of the (**b**) c-GST (111) and (**c**) c-GST (222) reflections. The thickness of the GST thin film was approximately 20 nm.

The next step of this study evaluates whether the a-GST/c-GST interface can be forced to propagate epitaxially towards the surface of the thin film by the accumulation of multiple laser pulses. That is, whether c-GST also forms epitaxially at an already existing a-GST/c-GST boundary. For this purpose, a further laser beam irradiation experiment is conceived. In detail, an amorphous GST thin film is deposited on a crystalline Si (111) substrate and crystallized by successive single ns-laser pulses at 21 mJ/cm^2^ fluence in order to incrementally increase the crystalline fraction of the thin film. After each applied laser pulse, a XRD pattern was recorded and these are depicted in Figure 5. In general, besides the Si (111) reflection, only the c-GST (111) and c-GST (222) reflections occur. The formation of (111) GST planes strongly suggest the crystallization of GST grains start from the Si (111) surface. Moreover, each laser pulse increases the crystalline fraction as can be seen by the incremental increase in peak intensity of the c-GST (111) and c-GST (222) reflections (Figure 5b,c). The increase in peak intensity is accompanied by a slight shift of the peak positions, which can be attributed to thermal strain introduced during the cool down process due to different thermal expansion coefficients of GST and Si. Therefore, these results verify that the increase in crystalline volume is based on epitaxial crystal growth starting at the c-GST/a-GST interface lying within the thin film. Consequently, based on these experiments, different reflectivity and conductivity states can be achieved by changing the volume ratios between amorphous and single-crystalline-like GST structures.

### 3.3. Interface-Assisted Phase Transitions from Layered Structures to Non-Layered Structure

Thus far, conventional crystalline-to-amorphous phase transitions have been studied by applying intense laser pulses to the as-deposited epitaxial GST thin films in order to initiate a melt quenching process leading to amorphization. Epitaxial GST thin films could, however, offer the opportunity to follow even small structural changes without initiating a complete amorphization. Therefore, epitaxial GST thin films were irradiated by ns-laser pulses at moderate laser fluences in order to assess whether even minor structural modifications can be triggered and, for example, whether a small thermal influx could lead to a randomization of the Ge/Sb/Vacancy sublattice in a vo-GST thin film.

**Figure 6:**
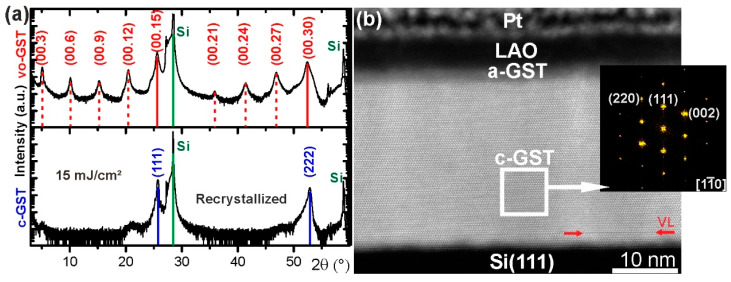
(**a**) XRD ω−2θ pattern of an epitaxial vo-GST thin film before (upper pattern) and after (lower pattern) irradiation with a single ns-laser pulse of a fluence of 15 mJ/cm^2^. The pattern of as-deposited vo-GST thin film shows superstructure reflections (vacancy layer peaks) marked by red dashed lines beside the main reflections marked by continuous red lines. The continuous blue and green lines mark (111) lattice planes in the c-GST and Si substrate, respectively. (**b**) HAADF-HRSTEM image of the laser irradiated thin film. Red arrows indicate a remaining VL. The inset shows FFT pattern, confirming the formation of the c-GST phase.

Figure 6a shows XRD ω−2θ patterns of an epitaxial vo-GST thin film before and after optical switching with a laser fluence of 15 mJ/cm^2^. Remarkably, despite the comparatively low laser fluence applied, significant changes in the XRD pattern are observed. In particular, a drastic reduction of the superstructure reflections attributed to the periodically spaced VLs becomes apparent. However, two pronounced peaks associated with c-GST (111) and c-GST (222) reflections remained. This indicates that the ns-laser irradiation not necessarily causes an amorphization process but rather a transition from one crystalline phase to another. In detail, the XRD results suggest an intracrystalline phase transition from vo-GST to c-GST.

Figure 6b shows a cross-sectional HAADF-HRSTEM image of the epitaxial GST thin film after applying the single ns-laser pulse. The TEM specimen was prepared from the laser irradiated area of GST thin film shown in Figure 6a. The image confirms the dominance of c-GST in the thin film and furthermore reveals a thin amorphous layer at the top of the thin film. Besides that, a VL of the initially vacancy ordered structure remained close to the Si substrate. This mix of phases probably leads to the slightly asymmetric reflections in the corresponding XRD pattern shown at the bottom of Figure 6a. The XRD and TEM results imply that the ns-laser irradiation leads to the annihilation of the VLs while the overall epitaxial nature remains. The observed phase transition might therefore be accomplished by only small movements of Ge/Sb atoms and vacancies, respectively. Furthermore, the spatial separation of the GST phases along the cross-section of the thin film (a-GST at the top, c-GST in the middle and vo-GST at the bottom) could be a hint for a temperature-induced transition process based on certain threshold values, since the laser irradiation leads to a pronounced temperature gradient along the cross-section of the thin film. Consequently, the high temperature at the top of the thin film leads to the formation of a-GST, whereas the lower temperature in the middle and at the bottom of the film might lead to the observed intracrystalline modification (or preservation) of the GST structure. Nevertheless, the remaining a-GST phase points out a recrystallization process via a transient molten phase, contradicting the mechanism of phase transition assumed from XRD data (see above).

**Figure 7:**
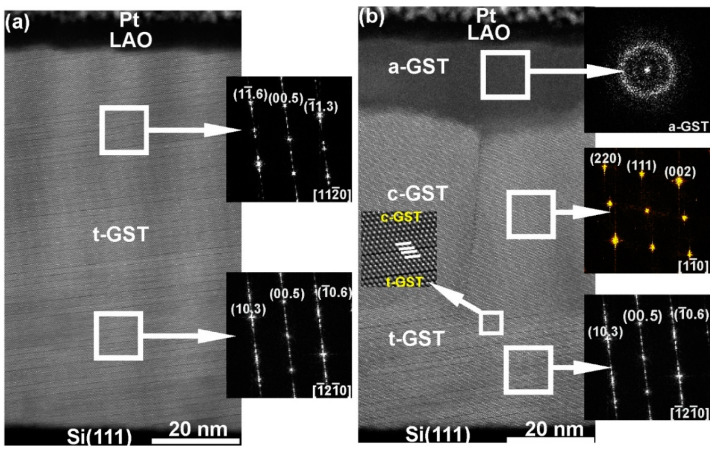
HAADF-HRSTEM images: (**a**) as-grown epitaxial t-GST thin film on Si (111) substrate with 6° miscut and (**b**) after laser irradiation by a single ns-laser pulse of a fluence of 23 mJ/cm^2^. The insets show FFT patterns calculated form rectangles marked in the images. The FFT patterns confirm the epitaxial relationship between the t-GST and the c-GST structures. At the bottom of the GST thin film in (**b**), the initial structure of epitaxial t-GST is preserved, whereas c-GST is formed in the middle of the thin film. At the top of the GST thin film, the material is melt-quenched into the amorphous phase. The insert in (**b**) gives a magnified image of the c-GST/t-GST interface, where white lines mark the (111) and (0001) lattice planes in the c-GST and t-GST structures, respectively.

In order to clarify the phase transition observed above (Figure 6), further studies concentrate on the optical switching of epitaxial t-GST thin films, which are structurally close to the vo-GST thin films. The intracrystalline transition from t-GST to c-GST structures would require huge structural changes associated with filling of vdW gaps by atoms and shifting of building units against each other. Such a transition is possible on long time scales only [41]. The t-GST thin film was grown on a Si (111) substrate with 6° miscut [42]. The (0001) planes of such thin films form inclined to the Si (111) substrate surfaces, resembling the surface topography of the substrates. Thus, in the case of interface-assisted crystal growth, the (111) planes of the recrystallized c-GST phase should be formed parallel to the (0001) planes of the as-deposited t-GST thin film. Figure 7 shows HAADF-HRSTEM images of a t-GST thin film before and after single ns-pulse laser irradiation with a fluence of 23 mJ/cm^2^. The film can be divided into three distinct regions (a-GST, c-GST and t-GST). According to the image contrast and corresponding fast Fourier transformation (FFT) patterns, the regions c-GST and t-GST represent cubic and trigonal crystalline structures, respectively. The FFT pattern calculated from region t-GST in Figure 7a reveals the as-grown t-GST phase, where the phase did not significantly change upon the laser irradiation. The t-GST phase is tilted by 6° with respect to the (111) Si interface. However, the interface between the t-GST and c-GST is parallel, where the (111) planes of c-GST develop parallel to the (0001) planes of t-GST, confirming the suggestion above. This indicates the crystallization of c-GST at the vdW gaps, where the t-GST acts as a template for epitaxial growth of the c-GST phase. Moreover, the crystallization from vdW interface indicates a temperature effect in the thin film induced by laser heating, where the phonon transport/heat transport interrupted at the vdW gaps.

The region a-GST in Figure 7b reveals an amorphous GST phase. The amorphization is due to a comparatively higher cooling rate in the top part of the thin film, which is a consequence of a high UV absorption of GST, leading to a higher temperature increase in the upper part of the thin film. Additionally, the thermal conductivity of a LAO layer (~12 W/mK) is larger than of t-GST (0.8 W/mK) itself, resulting in higher cooling rates at the top of GST thin film than inside of the thin film. Thus, the formation of the a-GST phase strongly indicates a melting and subsequent quenching process. In this particular case, the melting process is interrupted at the vdW gaps. Hence, the c-GST phase forms from a transient molten phase at the melt–crystalline interface upon the cooling process and crystallizes with an epitaxial relationship to the parent t-GST phase. These findings are particularly useful for determination of crystallization dynamics of epitaxial GST thin films. The combination of TEM (allows identification of thickness of the cubic phase, d in m) and pump-probe experiments (allows identification of time needed for the crystallization of c-GST, t in s) allow the determination of averaged crystallization rates (v = d/t) of the c-GST phase. By performing such experiments, in a recent work the authors reported crystal growth velocities lying between 0.4 and 1.7 m/s [22]. It was also found that the value of 1.7 m/s is the upper limit of such epitaxial GST thin film model systems, when amorphization is to be avoided [22].

### 3.4. Crystalline-to-Crystalline Phase Transitions via Structural Rearrangements

**Figure 8:**
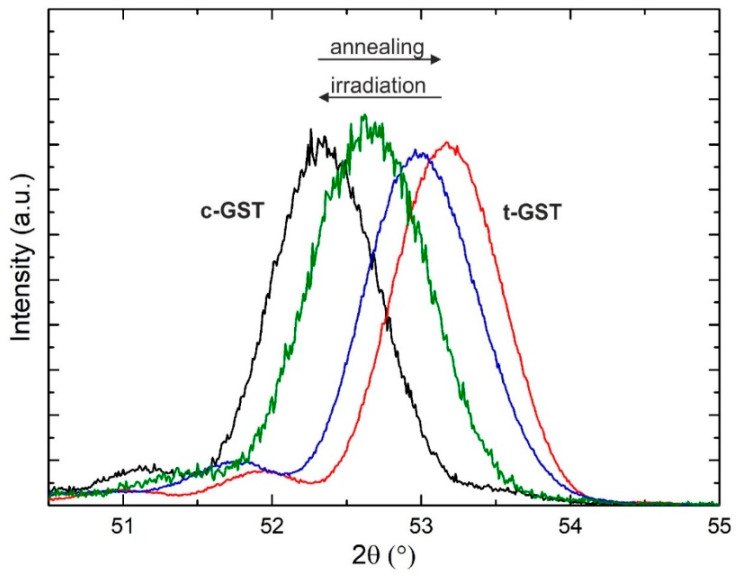
XRD pattern of the c-GST (222) and t-GST (00010) reflections of an epitaxial GST thin film after different steps of thermal treatment involving ns-laser irradiation as well as thermal annealing. The thickness of the GST thin film was approximately 15 nm (estimated from the Scherrer equation).

Ultimately, the above-presented results show a rapid transition from epitaxial vo-GST or t-GST thin films to epitaxial c-GST thin films. In this part of the study, the observed process is first subjected to a full switching cycle. It is known that the reverse process, i.e., the formation of vo-GST can be achieved by thermal heating [27,29,30,32]. This finally allows reversible phase transitions between epitaxial vo-GST and c-GST thin films. Such an intracrystalline switching cycle is demonstrated in Figure 8. Here, ω−2θ measurements at 2θ values in the range of the c-GST (222) and t-GST (00010) reflections (51.5° < 2θ < 54°) of an epitaxial GST thin film after treatments involving ns-laser irradiation and thermal annealing are shown. The measurements of Figure 8 reveal that while thermal heating at 250 °C for 30 min in the high-vacuum PLD chamber causes a transition from c-GST to t-GST (black to red XRD pattern), the ns-laser irradiation induces the reverse transition from t-GST back to c-GST. In particular, two successive ns-laser pulses are applied to gradually switch back to c-GST. After each laser pulse, the XRD pattern is recorded (represented by the blue and green pattern in Figure 8, respectively). Figure 8, therefore, demonstrates the reversibility of c-GST to t-GST phase transformations within an epitaxial framework of the crystal lattice. Interestingly, considering that there exists a viable reflectivity and conductivity contrast between the crystalline phases of GST alloys [26], these results could represent a promising switching pathway that offers the potential to reduce energy consumption as well as resistance drift of PCM devices by completely avoiding the amorphous phase during operation. Nevertheless, successful device implementation would still require detailed engineering studies on real PCM cells to determine the specific dose of energy and the corresponding timescales necessary to induce the desired transitions.

## 4. Conclusions

In conclusion, structural transitions in amorphous and layered epitaxial GST thin films (t-GST and vo-GST) induced by a single UV ns-laser pulse were studied by a combined approach using XRD and TEM analysis. Particularly, this work demonstrates different structural transformations, such as crystalline-to-amorphous phase changes, interface-assisted epitaxial crystallization, and phase transformations within crystalline phases. The amorphization of epitaxial vo-GST thin films and their recrystallization to c-GST thin films were achieved by applying optical pulses of high and low laser fluences, respectively. However, laser pulses of moderate fluences resulted in a single step structural transition to the c-GST phase. The phase was formed from a transient molten phase at the melt-crystalline interface upon the cooling process, providing access for identification of crystal growth velocities in epitaxial GST thin films [22]. It was also found that interface-assisted crystal growth led to the epitaxial formation of the c-GST structure. By varying the laser fluence, GST thin films with multiple phases and different amorphous to crystalline volume ratios can be realized in this approach [21]. This offers a possibility of multilevel data storage and realization of memory devices without or with very low resistance drift. Additionally, this work shows the reversibility of crystalline-to-crystalline phase transformations from c-GST to vo-GST followed by t-GST structures through structural rearrangements within an epitaxial framework. Moreover, this study demonstrates amorphization and crystallization of GST material by using only one UV laser with one single pulse duration and one wavelength, whereas the conventional amorphous-to-crystalline phase transitions are triggered by lasers with different pulse durations and number of pulses. Overall, the results of this work offer a new perspective on switching pathways in Ge-Sb-Te-based alloys and show the potential of epitaxial Ge-Sb-Te thin films for applications in phase change memory devices.
